# On Interlayer Stability and High-Cycle Simulator Performance of Diamond-Like Carbon Layers for Articulating Joint Replacements

**DOI:** 10.3390/ijms150610527

**Published:** 2014-06-11

**Authors:** Kerstin Thorwarth, Götz Thorwarth, Renato Figi, Bernhard Weisse, Michael Stiefel, Roland Hauert

**Affiliations:** 1 Empa, Swiss Federal Laboratories for Materials Science and Technology, Überlandstrasse 129, 8600 Dübendorf, Switzerland; E-Mails: renato.figi@empa.ch (R.F.); bernhard.weisse@empa.ch (B.W.); michael.stiefel@empa.ch (M.S.); roland.hauert@empa.ch (R.H.); 2 DePuy Synthes Companies of Johnson & Johnson, Luzernstrasse 21, 4528 Zuchwil, Switzerland; E-Mail: thorwarth.goetz@synthes.com

**Keywords:** diamond-like carbon, biomedical implants, adhesion, simulator testing, wear, coating, tantalum interlayer

## Abstract

Diamond like carbon (DLC) coatings have been proven to be an excellent choice for wear reduction in many technical applications. However, for successful adaption to the orthopaedic field, layer performance, stability and adhesion in physiologically relevant setups are crucial and not consistently investigated. *In vitro* wear testing as well as adequate corrosion tests of interfaces and interlayers are of great importance to verify the long term stability of DLC coated load bearing implants in the human body. DLC coatings were deposited on articulating lumbar spinal disks made of CoCr28Mo6 biomedical implant alloy using a plasma-activated chemical vapor deposition (PACVD) process. As an adhesion promoting interlayer, tantalum films were deposited by magnetron sputtering. Wear tests of coated and uncoated implants were performed in physiological solution up to a maximum of 101 million articulation cycles with an amplitude of ±2° and −3/+6° in successive intervals at a preload of 1200 N. The implants were characterized by gravimetry, inductively coupled plasma optical emission spectrometry (ICP-OES) and cross section scanning electron microscopy (SEM) analysis. It is shown that DLC coated surfaces with uncontaminated tantalum interlayers perform very well and no corrosive or mechanical failure could be observed. This also holds true in tests featuring overload and third-body wear by cortical bone chips present in the bearing pairs. Regarding the interlayer tolerance towards interlayer contamination (oxygen), limits for initiation of potential failure modes were established. It was found that mechanical failure is the most critical aspect and this mode is hypothetically linked to the α-β tantalum phase switch induced by increasing oxygen levels as observed by X-ray diffraction (XRD). It is concluded that DLC coatings are a feasible candidate for near zero wear articulations on implants, potentially even surpassing the performance of ceramic *vs.* ceramic.

## 1. Introduction

Owing to the aging process and related factors, some major joints of the human physiology are subject to decay during their lifetime and eventual replacement by artificial solutions. This mostly affects hip, knee, spine and shoulder, each of which generate more than one million replacement procedures each year. Generally, a solution retaining the articulation is preferred over a stiffening or fusion for the benefit of the patient and also for prevention of further decay of the neighboring structures; for example, articulating spinal disks (Total Disc Replacements, TDR) are of great advantage respective to the healing time, pain-management and less follow-up operations compared to arthrodesis [1,2].

To ensure a long lifetime of an artificial joint replacement it is crucial to know all possible failure mechanisms including wear mechanisms. These wear mechanisms differ depending on the joint materials and on the geometry of the implant. Furthermore, the question is not just one of reducing the amount of wear, but also how the size, shape, and surface chemistry of released wear particles differ among bearing surface combinations, since these factors may ultimately influence the biologic reaction and subsequent tendency for adverse body reactions (adverse local tissue response and systemic effects).

For metal-on-metal type designs, which possess a number of distinctive advantages over other material combinations [3], many potential issues can be addressed using low friction coatings for the bearing surfaces such as diamond like carbon (DLC). Aside from its success for wear reduction in the technical field [4], DLC coatings seem to be a potential solution to ion release and wear problems encountered with metallic articulating joint replacements [5]. DLC is well known for its chemical inertness, high hardness, low friction and high wear resistance [6,7]; yet the first aspect to be investigated when considering its use *in vivo* is the interaction between the DLC and its ambiance. For implants, the environment differs from a typical technical application by the presence of body fluid, which acts as a corrosive medium. This opens the possibility for corrosion-assisted failure mechanisms as shown in previous publications [8,9]. Specifically, it was found that a crack can propagate along the reactive DLC/metal interface in body-like environment and that this propagation can be explained by the laws of stress-corrosion cracking (SCC) [10,11,12]. Furthermore, the potential of proteins to clog e.g., pinholes or small cracks can generate crevice conditions [13]. Another issue with application of DLC coatings is securing adhesion to the substrate material owing to its high compressive stress; this is typically facilitated by means of a reactive interlayer [14]. A detailed overview of DLC coatings in the field of medical applications is given by several groups [4,15,16,17]. The aim of this work is to investigate the qualification of DLC coatings for long-term application in bearing implants using the example of lumbar spinal disks regarding mechanical stability, corrosion resistance as well as defect tolerance and third body wear.

## 2. Results and Discussion

**Figure 1:**
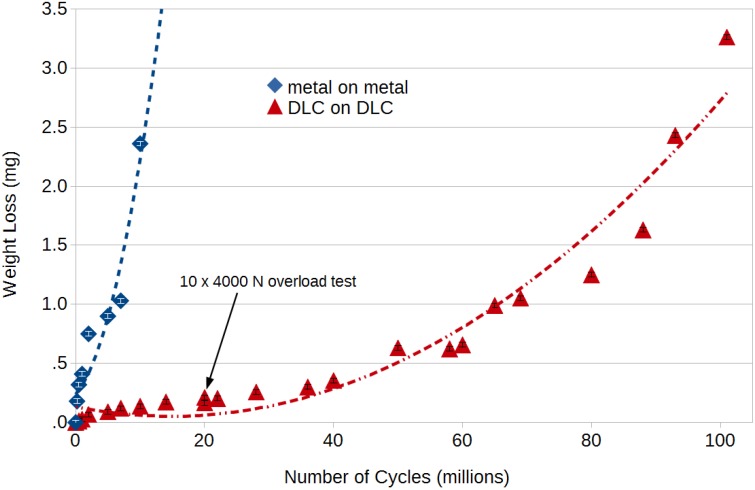
Gravimetrically measured weight loss over 100 million cycles (simplified simulator setup) with comparison to an uncoated (metal-on-metal) pair.

The weight loss curve of the convex part of a DLC coated cobalt chrome molybdenum (CCM) spinal lumbar disk over the number of articulating cycles up to 101 million cycles of articulation is given in Figure 1. For comparison the weight loss for uncoated CCM spinal disks is shown up to 10 million cycles. Calculating the weight of a 4 μm thick DLC layer with an estimated area of 1 cm^2^ gives a total mass loss of 1.2 × 10^−3^ g, corresponding to a loss of the total layer volume after 70 million cycles. This contradicts the observation that the layer still looked largely undamaged after this cycle number. In direct comparison to an uncoated metal-on-metal part, the generation of high wear on the latter following a run-in phase up to 7 million cycles is evident. This effect has been linked to roughening of the uncoated CCM surface [18] by third-body wear particles and consequent deterioration of the lubrication regime, which also correlates to observations on explanted metal-on-metal joints [19]. In contrast, the gravimetry results on the coated parts show that the surface integrity is retained up to high cycle numbers as also visible to optical inspection. Also, static loads applied to the DLC coated wear couple at 20 million cycles (4 kN, 10 repeats) did not affect or induce any significant increase in wear.

**Figure 2:**
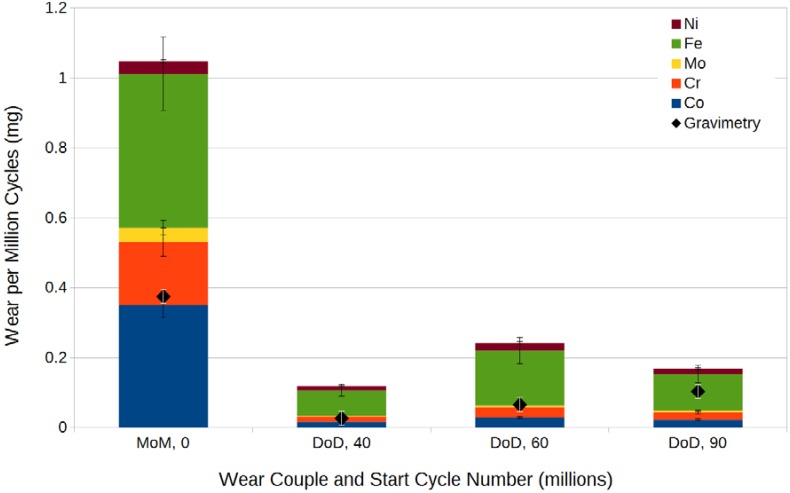
ICP-OES (inductively coupled plasma optical emission spectrometry) measurement results for the testing fluid in comparison to the gravimetry results.

To corroborate the gravimetry results, ICP-OES was performed to analyze the wear fluid ion concentrations at selected intervals as presented in Figure 2. It is evident that large parts of the detected metal debris stem from the simulator setup and sample fixture, which were stainless steel clamps tightened around protrusions on the convex wear part backside, while the concave part was permanently pressed and locked into the fixture. Comparing the specific wear to the gravimetrically measured wear, the results are in good quantitative agreement especially for the mid-range DLC-on-DLC couple. Noting that the gravimetrically determined weight loss only accounts for the convex part and the optically intact coating, the preliminary hypothesis may be drawn that the observed wear largely originates from fixture-sample backside articulation, and the gravimetry curve (Figure 1) may be corrected accordingly.

**Figure 3:**
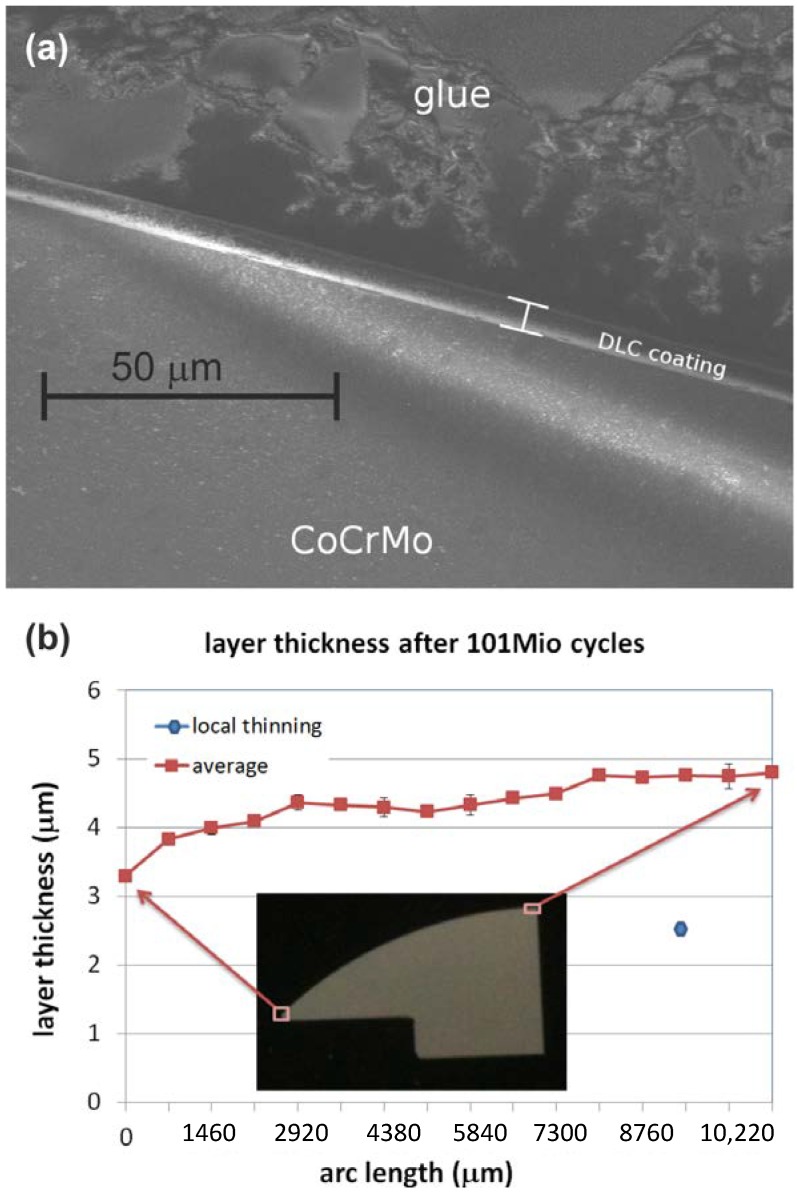
(**a**) SEM (scanning electron microscopy) image of cross-section of the convex implant after 100 million cycles; (**b**) Resulting layer thickness distribution derived from the cross sections.

In cross-section SEM view (Figure 3a), this hypothesis is solidified. Layer thickness measurements taken on all 250 cross-section images indicate no large-area deviation from the part’s original 4 µm layer thickness (Figure 3b), with only few individual instances of local thinning detected. Factoring in the low specific weight of carbon wear, the weight loss measured by gravimetry cannot be explained by wear on the articulating surfaces and must be attributed to wear of the sample backside with the sample fixture instead. It can therefore be concluded that DLC coatings on matched and polished metal articulations represent a very low wear tribocouple, which was also observed in the case of hip joints [20].

Another interesting aspect of high cycle number *in vitro* testing is the evolution of local defects. As observed on several occasions and also shown elsewhere [9,12], growth of local defects can lead to an avalanche-like delamination once a defect size threshold is exceeded and the defects start to interact including third-body wear mechanisms. A successful coating thus has to tolerate local defects that may arise even during the coating process by coverage with dust particles or pull-out of loose grains from the substrate material.

**Figure 4:**
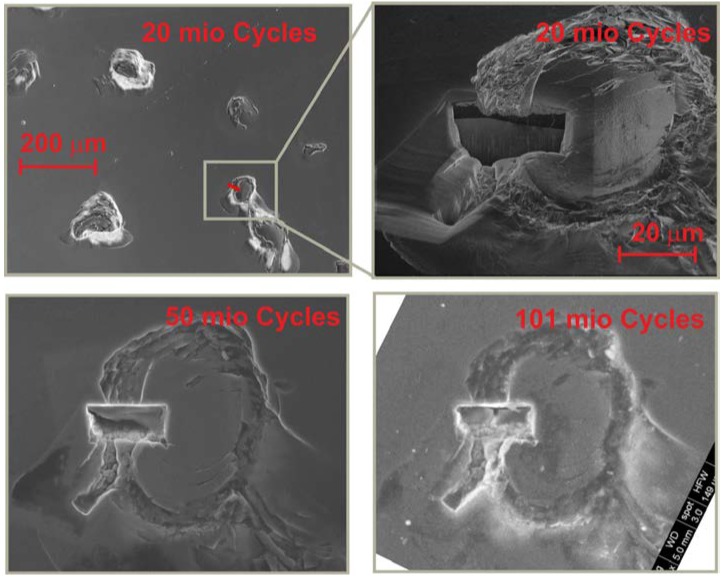
Defect evolution on a selected defect site after 20, 50 and 100 million cycles.

Figure 4 shows the changes to an exemplary defect observed at 20, 50 and 101 million articulation cycles on the convex part of a simulator articulation pair. This group of defects was originally observed early after 3 million cycles of articulation. A focused ion beam (FIB) cut was placed through the defect’s edge to gain insights on eventual interfacial cracking. However, no evolution of the defect size or shape at the given cycle numbers was observed, meaning the selected interface is tolerant towards small failures.

**Figure 5:**
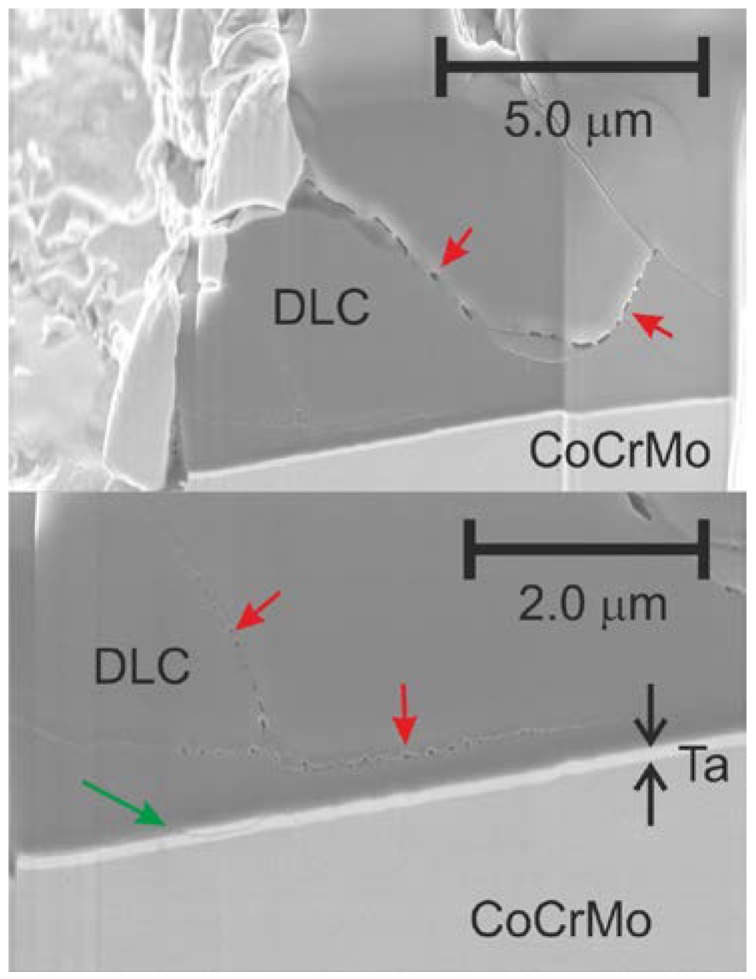
Focused ion beam (FIB) cross section on a defect edge after 100 million cycles. Red arrows: “Bubble-like” failures, green arrow: Crack exiting the interface.

Figure 5 further illustrates the layer behavior after high cycle testing (101 million cycles) in several FIB cut cross sections (center region of the convex part). In contrast to a mechanically or chemically weak interface, the present cracks do not propagate along the DLC-substrate interface, instead deviating into the DLC layer. Although the general presence of cracks cannot be avoided due to the eggshell effect, this behavior might help to keep the defects more localized and not lead to large-scale delamination.

An additional feature found in Figure 5 is a “bubble-like” form of the cracks in cross-section. This morphology has not been observed at low cycle numbers and bears resemblance to fish grate-like surface defects occasionally observed on top-view images. Potential explanations may be a form of fatigue of the DLC coating, however more detailed studies are required for further discussion.

**Figure 6:**
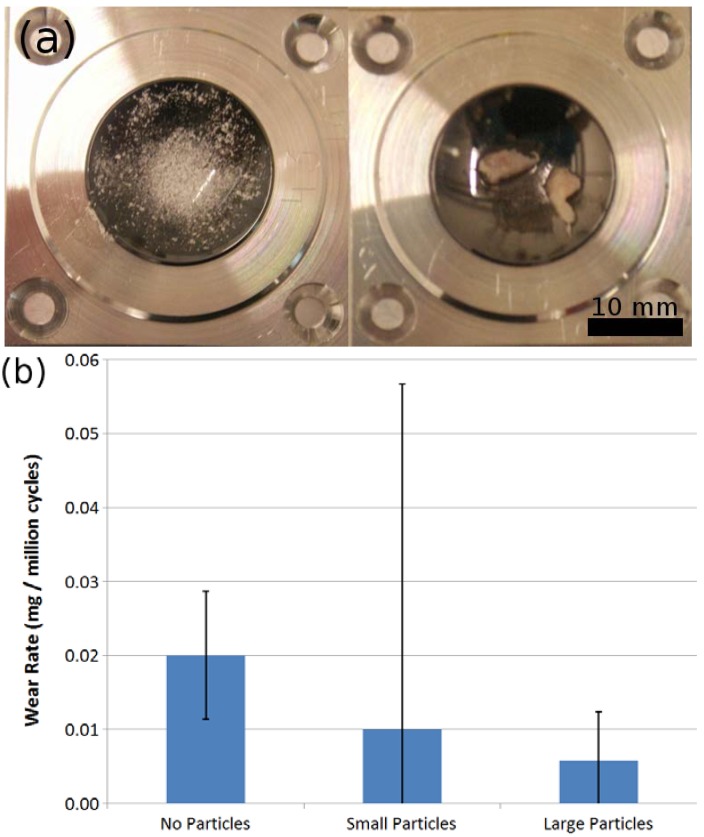
(**a**) Third-body wear particles and (**b**) resulting wear rate on DLC/DLC pairs.

At 28 million cycles of articulation of an analogous sample pair, cortical bone powder was added into the tribocontact to simulate a third-body wear situation. The resultant wear values are illustrated in Figure 6. Both optical inspection and gravimetric analysis did not indicate a significant wear increase or roughening due to addition of third body wear particles. It was noted that both variants of wear particles were rapidly expelled from the contact, with the larger particles being ground into smaller fragments. Further investigations are needed to check for adverse effects of other potential third-body wear materials like bone cement (e.g., Polymethylmethacrylate (PMMA)).

For investigations of potential failures of the Ta interlayer due to oxygen contamination during deposition, several possible failure mechanisms are to be considered:

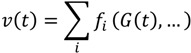
(1)
where *v*(*t*) delamination speed (limit: lifetime requirement), *f_i_* (*G*(*t*)*,…*) failure mechanisms, *G*(*t*) = *G_intrinisic_ + G_variable_* (*t*) interface energy. With examples *f_cc_* (*G*(*t*)*,…*) *= A* crevice corrosion-type failure, *f_scc_* (*G*(*t*)*,…*) *=* (*G*(*t*) *− G_scc_* )^*B*^ stress-corrosion cracking type failure, 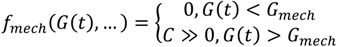 instant mechanical type failure.

Particularly, these mechanisms can be attributed to pure mechanical (static and fatigue-based), stress-corrosion-based and pure corrosive effects (e.g., crevice corrosion), all of which were found relevant in recent publications [8,9,12,13]. It should be noted that the failure mechanism can change through the implant lifetime, *i.e.*, one mechanism can act as a “lead-in” for the other. Depending on the individual initiation thresholds, this can greatly complicate failure analysis for long-term implants targeting to cover all possible failure paths.

As of today, not all failure mechanisms outlined above can be tested effectively. No accurate way to accelerate the process is known especially for the corrosion-dominated mechanisms; for these, a combination of long-term observation and microscopic analysis (FIB/SEM) is presently required.

**Figure 7:**
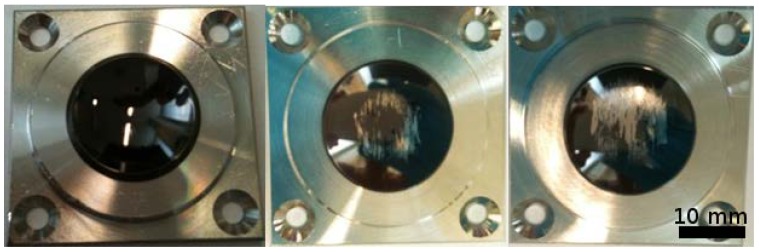
Delamination observed on coated articulation pairs (concave parts) with increasing interlayer oxygen contamination. **Left**: *R* = 0, 80 million cycles; **center**: *R* = 5 × 10^−4^, 100,000 cycles; **right**: *R* = 6.4 × 10^−2^, 100,000 cycles.

For the present interlayer system (CCM/Ta/DLC), defined oxygen contamination levels were chosen as detailed in Table 1 to obtain failure limits for purely mechanical failure and stress-corrosion cracking. For the simulator tests, rapid failure even for low oxygen contamination (*R* ≥ 5 × 10^−4^) resulted in cycle numbers <100,000, with worsening delamination pattern at higher concentrations (Figure 7). In contrast, several uncontaminated articulation pairs did not show any signs of delamination up to 20 and even 101 million cycles.

**Table 1:** Implants coated in this study and resultant interlayer stability. Only samples with *R* = 0 were found to be stable up to high cycle numbers in the simulator tests, whereas only samples with *R* = 6.4 × 10^−2^ (red) exhibited stress-corrosion-cracking behavior.

*R* = O_32_/Ar_40_	0		5 × 10^−4^	5 × 10^−3^	1 × 10^−2^	3× 10^−2^	6.4 × 10^−2^
Ta interlayer	Sample No.	cycles	P42	P37	P36	P35	P32
	P03	33 million					
	P07	101 million					
	P43	29 million					
	P45	20 million					
	P33	25 million					
	P34	25 million					

**Figure 8:**
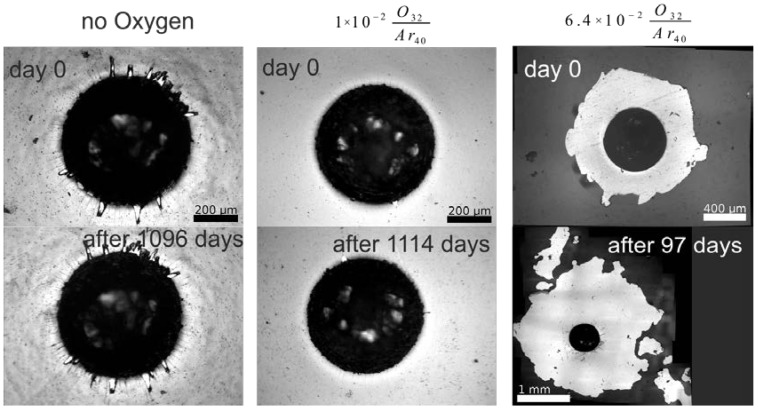
SCC (stress-corrosion cracking) test (delamination around indents) placed on DLC-coated reference samples with increasing oxygen interlayer contamination.

Stress-corrosion cracking tests on the oxygen-contaminated interlayers were performed by 1500 N Rockwell indentation, immersion in phosphate buffered saline solution and following the time-dependent delamination radius as detailed earlier. The stress-delamination speed dependence allows for modeling of the SCC process for a given materials system. In the present case, the onset of SCC was found at much higher oxygen contamination levels compared to mechanical failure, leading to very quick crack propagation and failure above this level (Figure 8).

Finally, concerning the potential for purely corrosion based failure (crevice corrosion, CC), the FIB cross sections on the 101 million cycle defect edges may be reviewed for indications of a dissolving (blunt) crack. Since these samples were immersed into physiologically relevant testing fluid for more than 3 years, an upper limit for CC based dissolution rate may be deducted. No indications of crack propagation were found in these analyses. Hence it may be concluded that crevice corrosion does not play a dominant role in the potential *in vivo* failure mechanism of the Ta interlayer system, in contrast to Si based interlayers [12,13].

**Figure 9:**
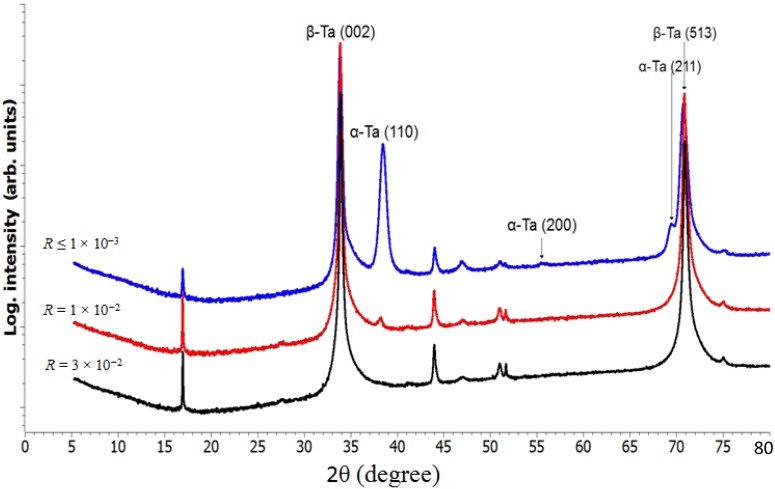
X-ray diffractograms of the Ta interlayer structure with respect to the oxygen contamination level (O_32_/Ar_40_ current ratio *R*).

In summary, purely mechanical failure seems the predominant effect among the tested failure modes for the presented interlayer system (Ta). To elucidate the source of this behavior, X-ray diffraction was performed for the Ta interlayer structure on three distinct contamination levels (Figure 9). Changes are visible in particular to the α-Ta (110) and β-Ta (002) peaks, with the α-Ta peak disappearing at higher contamination levels. Considering that the structure factor of β-Ta (002) exceeds the α-Ta (110) by a ratio of 7.01 [21], the proportion of the α-Ta phase is relatively minor. However, it must be noted that the amorphous and nanocrystalline Ta content of the layer is unknown, that the β phase is documented as brittle in literature [22], and that oxygen is known to stabilize the β phase in Tantalum [23,24]. Judging from the increase in absolute intensity of the β phase peak between the three contaminations investigated, the absolute amount of X-ray detectable β-Ta is found to increase by a factor of 2.75 between the highest and lowest dataset. As a hypothesis, it might therefore be suggested that oxygen contamination leads to high β content of the Ta interlayer and thus enables mechanical failure.

## 3. Experimental Section

Four µm thick DLC layers were deposited on mirror polished high-carbon biomedical CoCr28Mo6 (CCM) implant alloy using radio frequency (13.56 MHz) plasma activated chemical vapor deposition (PACVD) with acetylene (C_2_H_2_) as a process gas. As adhesion promoting layer a 90 nm Tantalum interlayer was deposited *in situ* by magnetron sputtering using a pure Tantalum target (5 N) without interruption of the plasma discharge between process steps. Prior deposition the CCM substrates were ultrasonically cleaned in an acetone-ethanol mixture and additionally presputtered in an argon discharge (19 sccm Ar flow; −600 V RF bias). The gas flow for Acetylene was fixed at 24.0 sccm (2.5 Pa) and for sputtering a gas flow of 2.1 sccm (0.5 Pa) Argon was used. The target was presputtered during the last minutes of the precleaning process of the sample. For all experiments a base pressure of <1 × 10^−5^ Pa was established before process initiation. A more detailed description of the DLC deposition process can be found in [25].

The microstructure of the DLC layers was determined with a FEI NovaNanoSEM 230 and a Hitachi S-4800 scanning electron microscope. For the cross-sections, a focused ion beam instrument (FIB-Dual Beam FEI STRATA DB235) was used. 

To analyze wear behavior, coatings were deposited on ball-on-socket type lumbar spinal disk replacement implant prototypes made of CCM with a radius of 14.5 mm. A simplified spinal simulator setup with reference to ASTM F2423-05 was chosen [18], featuring a constant perpendicular load of 1200 N and one degree of motion, which was applied alternatingly in human lumbar spine lateral (+/−2°) and flexion-extension (+6°/−3°) mode. These ranges were chosen in accordance with ISO 18192-1. For the change of motion mode, samples were rotated +/−90° after 0.2, 0.5, 1, 2, 5 and 10 million total cycles and every 10 million cycles at successive intervals. The articulation frequency was 3 Hz. The sample stage was kept immersed into 37 °C 30 g/L protein-containing wear testing fluid (Hyclone^®^, Cat. No. SH30856.04, Thermo Fisher Scientific, Logan, UT, USA), which was stabilized with anti-fouling agents (NaN_3_, protease inhibitors) and periodically exchanged. Ultrapure water was refilled to compensate for the evaporation, preventing volume and concentration changes of the lubricant during the tests. Running simulator tests in non-protein media like Phosphate Buffered Saline (PBS) would lead to deviations both in lubrication mode and corrosion-assisted failure characteristics and is hence not recommended [18,26].

In the course of dismounting the sample for a +/−90° rotation on the simulator stage the samples were cleaned according to ISO 14242-2 and the weight loss was determined with weight measurements (ES 225SM-DR, Precisa, Dietikon, Switzerland; AE-163, Mettler Toledo, Greifensee, Switzerland; resolution 0.01 mg). On selected defect sites, Focused Ion Beam (FIB) cross sections were cut on a FIB-Dual Beam instrument (FEI STRATADB235) using a gallium ion beam. Additional optical investigations were performed with a Philips XL30 ESEM-FEG scanning electron microscope (SEM) equipped with an EDX (energy dispersive X-ray) detector.

Exchanged simulated wear testing fluid was itemized by thermal cracking in a 65 vol. % nitric acid/30% hydrogen peroxide mixture at a temperature of 190 °C. Following thermal cracking, all samples were optically clear. The Co, Cr, Mo, Fe and Ni content of the samples was measured using ICP-OES referencing certified standards.

Following 101 million cycles of testing, one convex sample part was cut into 90° sectors by a diamond saw and analyzed for remaining coating thickness in cross-sections using SEM (FEI NovaNanoSEM 230). 250 cross-section images were taken to gain an overview over the thickness distribution.

To corroborate the high-cycle simulator tests, additional studies with hard third-body wear particles were performed. For this, coarse and fine-grained cortical bone was machined from porcine ribs and inserted into the articulation couple at 28 million cycles. Gravimetrical measurements and optical inspection were performed to identify potential layer failures or increases in wear volume.

The tolerance of the tantalum interface towards contaminations during the deposition process was investigated. For this, a defined oxygen amount with respect to the process gas pressure was admitted to the chamber during the interlayer growth by means of an oxygen leak valve. The level of oxygen was monitored and set via the *m*/*q* = 32 to *m*/*q* = 40 current signal ratio (*R*), *i.e.*, *versus* the detected argon current using a mass spectrometer (SPM 200, Pfeiffer Vacuum, Zurich, Switzerland) immediately prior to starting the RF discharge for sputter cleaning. The targeted I (O^2+^/Ar^+^) current ratios varied from 0 to 6.5 × 10^−2^. The structure of the adhesion promoting Tantalum interlayer was determined by X-ray diffraction (Bruker D8, Bruker, Karlsruhe, Germany) using monochromated Cu Kα radiation in Bragg-Brentano configuration. Stress corrosion cracking (SCC) tests of the interfaces involved were determined as described in [11]. A standard Ernst NR 3R Rockwell indentation setup was used to induce the delamination of the DLC coatings via the plastic deformation of the CCM substrates. The diamond Rockwell tip with a 120° cone was pressed into DLC coated substrate with a load of 1.5 kN for 10 s.

After indentation the samples were immersed in 0.01 M phosphate buffered saline (PBS) solution (Sigma Aldrich, Buchs, Switzerland). All fluids were maintained at a constant temperature of 37 °C. A Müller (Mueller-Optronic, Erfurth, Germany) metallographic microscope equipped with a Premiere^®^ MA88-300 CCD camera was employed to investigate the time dependent delamination of the thin films in corrosive media.

Finally, to test defined contamination interlayers for mechanical failure, a series of corresponding articulation pairs was prepared and run in the spinal simulator setup described above until delamination was observed.

## 4. Conclusions

In this work, investigations on the stability of Tantalum interlayers for DLC coated articulating implants were presented. It was found that uncontaminated (with respect to oxygen) Tantalum interlayers give excellent adhesion stability to DLC coatings on CCM articulations, with little to no noticeable wear detected after 101 million cycles in a simplified lumbar spine simulator setup. Furthermore, it was found that overloading and third-body wear situations with bone particles do not effect significant coating failure, and that local defects do not exhibit growth even at prolonged *in vitro* testing. Therefore, the wear characteristic of a DLC coated metal-on-metal articulation fulfils even extended lifetime requirements for joint replacements.

Regarding the Ta interlayer stability against coating process contamination, mechanical failure was analyzed to be predominant for the presented materials system, with very low contamination levels leading to early cycle failure in the simulator. Adding structural characterization results, this failure was preliminarily linked to β Ta evolution induced by oxygen doping during the interlayer growth process and the corresponding embrittlement. Other failure modes (like SCC) were found to be present but requiring much higher oxygen contamination levels to exceed the activation threshold. It is therefore concluded that amorphous/α phase Ta interlayers represent a viable solution for securing DLC adhesion on CCM orthopaedic implants, given that tight contamination control is maintained.
